# Reinforced Defenses: *R*-Genes, PTI, and ETI in Modern Wheat Breeding for Blast Resistance

**DOI:** 10.3390/ijms262010078

**Published:** 2025-10-16

**Authors:** Md. Motaher Hossain, Farjana Sultana, Mahabuba Mostafa, Imran Khan, Lam-Son Phan Tran, Mohammad Golam Mostofa

**Affiliations:** 1 Department of Plant Pathology, Gazipur Agricultural University, Gazipur 1706, Bangladesh; hossainmm@gau.edu.bd (M.M.H.); mahabubamimt@gamil.com (M.M.); 2 College of Agricultural Sciences, International University of Business, Agriculture and Technology, Dhaka 1230, Bangladesh; farjana1s@iubat.edu; 3 Department of Chemistry, State University of New York College of Environmental Science and Forestry, Syracuse, NY 13210, USA; ikhan@esf.edu; 4 Department of Plant and Soil Science, Institute of Genomics for Crop Abiotic Stress Tolerance, Texas Tech University, Lubbock, TX 79409, USA

**Keywords:** *Magnaporthe oryzae* pathotype *Triticum* (MoT), transcriptomics, marker-assisted selection (MAS), CRISPR/Cas, plant immunity

## Abstract

Wheat blast, caused by *Magnaporthe oryzae* pathotype *Triticum* (MoT), poses a major threat to wheat (*Triticum aestivum*) cultivation, particularly in South America and Bangladesh. The rapid evolution and spread of the pathogen necessitate the development of durable and broad-spectrum resistance in wheat cultivars. This review summarizes current insights into the multi-layered defense mechanisms of wheat, encompassing resistance (*R*) genes, pattern-triggered immunity (PTI), and effector-triggered immunity (ETI) against MoT. The *R*-genes provide race-specific resistance through ETI, while both ETI and PTI are required to form integral layers of the plant immune system that synergistically reinforce host defense network. Recent advances in genomics, transcriptomics, and molecular breeding have facilitated the discovery and deployment of key *R*-genes and signaling components involved in PTI and ETI pathways. Integrating these immune strategies through gene pyramiding, marker-assisted selection (MAS), and genome editing offers a promising route towards enhanced and durable resistance in hosts. Harnessing and optimizing these multilayered immune systems will be pivotal to securing wheat productivity amid the growing threat of wheat blast.

## 1. Introduction

Numerous pests and pathogens, including fungi, oomycetes, viruses, nematodes, and bacteria, can contact plants; however, only specific interactions can lead to plant diseases. Yet, plant pests and diseases account for an annual loss of about 26% of the world’s crop production [1]. Minimizing yield losses from plant diseases is thus crucial, especially in light of growing human populations and shrinking arable land resulting from climate change, erosion, and water scarcity [2]. To guarantee the food and nutritional safety for the growing global population, the sustained production of major cereals, including wheat, is crucial. Along with rice (*Oryza sativa*) and maize (*Zea mays*), wheat makes up a considerable part of the typical human diet and contributes roughly 50% of the global food energy. As a single crop, wheat (*Triticum aestivum*) alone accounts for 20% of the global food calories and protein [3], making it one of the world’s most widely consumed food crops.

**Figure 1:**
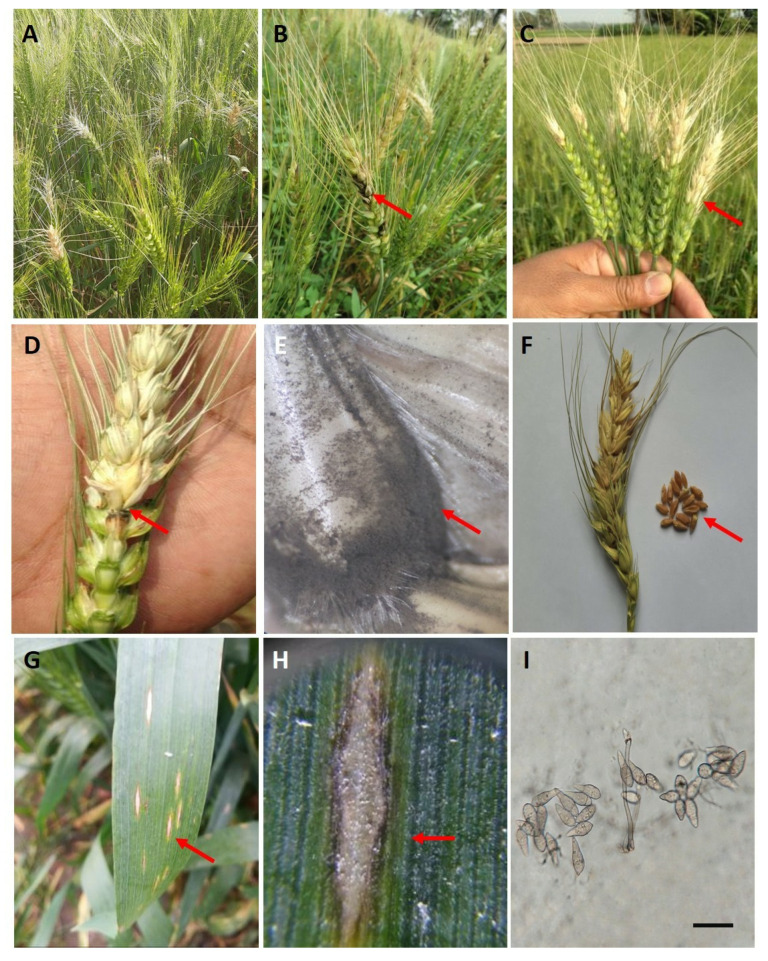
Typical wheat blast disease symptoms and signs caused by *Magnaporthe oryzae* pathotype *Triticum* (MoT)**.** (**A**) A wheat field showing characteristic signs of blast infection with partially bleached spikes; (**B**) Infected wheat plants showing wheat blast symptoms on wheat heads, with dark-colored infection points. (**C**) Wheat spikes exhibiting partial bleaching and disruption of spikelet development; (**D**) Severe infection of the wheat rachis by MoT, leading to necrosis, blockage of nutrient flow, and spikelet death. (**E**) Infected glume with dark-gray sporulation of the fungus MoT. (**F**) Severely shriveled or wrinkled wheat grains from the blast-affected spike. (**G**) Typical elongated or elliptical lesions on wheat leaves. (**H**) A typical elliptical lesion with white to tan centers and a reddish-brown margin on a mature leaf. (**I**) Two-septate hyaline to pale gray-colored pyriform conidia of MoT under a compound microscope (magnification 400×). Red arrows indicate characteristic disease symptoms or fungal structures associated with wheat blast. (**G**–**I**) were taken from [5]. Scale bar  =  20 μm.

However, wheat is continually threatened by new infectious diseases that are harmful in both developed and developing countries. One such disease is wheat blast, also known as “brusone,” caused by the *Magnaporthe oryzae* pathotype *Triticum* (MoT). The disease has emerged as a grave biological menace to food security in South America, Bangladesh, and Zambia [4]. Wheat blast is relatively new in most wheat-growing areas and has presumably developed as a result of a sequence of “host jumps” from a related grass species [5]. The formidable MoT strain is prevalent in humid subtropical climates and typically infects the lower or upper rachis, disrupting spike formation. This damage can result in partial or complete death of the spikes, leading to shriveled seeds or no grain, respectively (Figure 1A–F) [4]. At the site of infection on the rachis or glumes, dark-gray or black sporulation of the fungus can be observed (Figure 1E). Microscopic examination of the rachis or glumes may also reveal the presence of septate, pyriform conidia of MoT (Figure 1I). In highly susceptible cultivars, severe MoT infection under favorable disease conditions can have catastrophic consequences, resulting in up to 100% crop loss [5]. Given its destructive potential, an emerging and increasingly widespread outbreak of wheat blast poses a significant risk to global food security.

Managing wheat blast is highly challenging as the most readily available approaches often fail to provide a sufficient degree of control. While crop rotation is a key agricultural practice that helps decrease pathogen inoculum levels in the field, these inocula can still persist on weeds and different crop hosts [6]. Biological agents, such as *Bacillus methylotrophicus*, have been proposed as an effective alternative for controlling wheat blast [7], but their efficiency has not been proven in the field. The use of chemicals has been frequently suggested as a means to control wheat blast. However, during periods of significant disease pressure, fungicides have been shown to be impractical for suppressing wheat blast [8,9]. Furthermore, resistance to the most commonly used triazole and strobilurin fungicides has been reported [10,11]. Under these circumstances, utilizing host resistance is the most preferred method for achieving sustainable control of wheat blast [12]. However, most cultivated wheat varieties are susceptible to blast disease, necessitating the development of blast-resistant cultivars through modern wheat breeding strategies.

Plant disease resistance comprises complex, multilayered networks [13]. Upon pathogen attacks through microbial- or pathogen-associated molecular patterns (MAMPs/PAMPs), plants initially mount pattern-triggered immunity (PTI), followed by effector-triggered immunity (ETI), in which host resistance (R) proteins recognize pathogen effectors and deploy robust, rapid defense responses [14]. These innate and adaptive mechanisms operate synergistically to reinforce host protection and eliminate the invading threat. Recent advances in genomics, molecular breeding, and gene editing have enabled the identification and incorporation of *R*-genes, PTI-associated factors, and ETI-related mechanisms. By targeting these defense mechanisms in modern wheat breeding programs, breeders can develop wheat varieties with durable and broad-spectrum resistance to wheat blast. To formulate strategies to utilize multiple defense layers against the deadly wheat blast pathogen, this review explores the roles of *R* genes, PTI, and ETI in wheat defense against blast. It also discusses their potential integration into modern wheat breeding programs. Finally, the review addresses the challenges and future directions for achieving long-lasting resistance in wheat against MoT.

## 2. Biology of the Wheat Blast Pathogen

**Figure 2:**
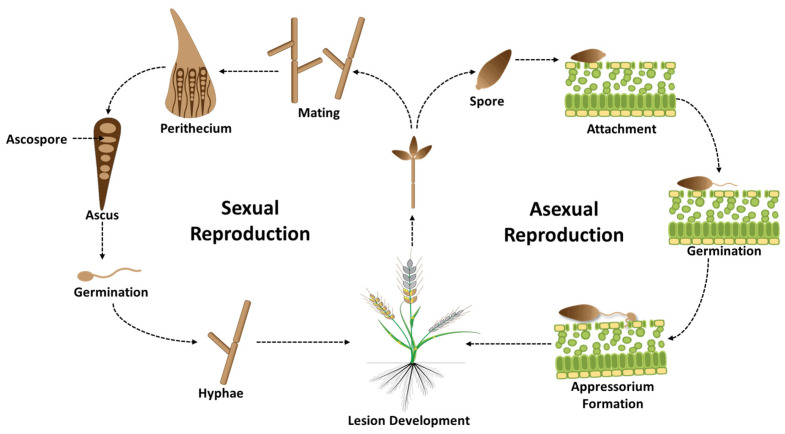
Life cycle of *Magnaporthe oryzae* pathotype *Triticum* (MoT), the causal agent of wheat blast. The pathogen exhibits both asexual and sexual reproduction, enhancing its adaptability and evolutionary potential. During asexual reproduction, pyriform-shaped conidia germinate on moist host surfaces, develop appressoria, and penetrate the cuticle to colonize host tissues. Sexual reproduction, though infrequent, involves the formation of microconidia and ascospores, contributing to genetic diversity and the emergence of new virulent strains.

*M. oryzae* (syn. *Pyricularia oryzae*), formerly known as *M. grisea*, is a species complex infecting more than 50 grasses, including wheat, rice, barley, oats, ryegrass, millet, and crabgrass [15,16]. Within this complex, distinct host-specific lineages exist, with the wheat-infecting population recently separated as *M. oryzae* pathotype *Triticum* (MoT) [6]. The fungus reproduces both asexually and sexually (Figure 2). Asexual reproduction occurs via pyriform, three-celled conidia that are hyaline to pale gray and serve as the primary source of inoculum; these conidia germinate on hydrophobic surfaces and form melanized appressoria that generate high turgor pressure to penetrate host tissues [12]. Sexual reproduction, although less frequent in nature, produces perithecia containing four-celled ascospores, while some strains also form a Phialophora-like anamorph that produces microconidia of uncertain ecological role [17,18]. Both conidia and ascospores can initiate infection under favorable conditions, and penetration failures on non-adapted hosts often elicit hypersensitive-like responses [19]. MoT is a hemibiotrophic fungus that initiates infection with a biotrophic phase, colonizing living host cells before transitioning to a necrotrophic stage that kills tissue to extract nutrients [12]. Optimal development and spread of MoT occur under warm (25–30 °C), humid conditions with extended leaf wetness, which favor conidial germination, sporulation, and epidemic outbreaks, particularly during flowering [5]. Wind and rain-splash contribute to local and long-distance dispersal, underscoring the pathogen’s capacity for rapid epidemic expansion [6,20]. Between cropping seasons, MoT survives in crop residues, alternative hosts such as *Digitaria* spp., and contaminated seeds, which facilitate both local survival and long-distance dispersal [21,22,23].

## 3. Wheat Blast: An Emerging Threat

**Figure 3:**
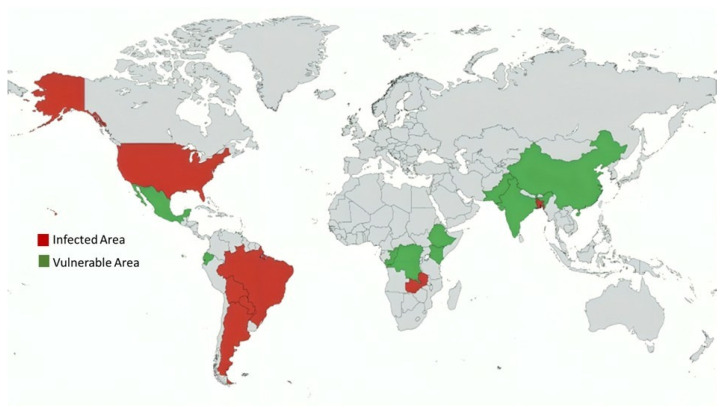
Global distribution and risk zones of wheat blast disease. Countries with confirmed outbreaks are shown in red, while those considered highly vulnerable due to favorable climatic and agricultural conditions are shown in green. The causal pathogen *Magnaporthe oryzae* pathotype *Triticum* (MoT) was first reported in Brazil and is now endemic in several South American countries. A major intercontinental outbreak in Bangladesh highlighted the serious threat to South Asian wheat production. More recently, the detection of wheat blast in Zambia has raised concerns over the potential spread to other wheat-producing regions in Africa, including Ethiopia, Kenya, and the Democratic Republic of the Congo. The figure is constructed using data from [4,5,26,27,28,29,30,31].

Wheat blast is among the most fearful and persistent wheat diseases in recent decades. After its first official account in 1985 in Paraná, Brazil [24], the disease subsequently spread to other wheat-growing states of Brazil (Table 1), impacting around 3 million hectares in the early 1990s. A historical context of wheat cultivation in Mato Grosso do Sul may help explain its catastrophic impact on wheat productivity in Brazil. In 1987, wheat cultivation in Mato Grosso do Sul reached a peak of 428,000 hectares, but by 2016, it had decreased by 95%, to around 20,000 hectares, primarily due to wheat blast epidemics [25]. The spreading of tropical wheat in the Cerrado region has also been prevented due to the wheat blast. The disease also infiltrated wheat agroecosystems beyond Brazil, including Bolivia, Paraguay, and Argentina, and persistently jeopardized the viability of wheat cultivation in the region. In 1997, Bolivia experienced a 69% reduction in wheat yield due to a severe blast epidemic [26]. Various factors, such as climate change, the continued cultivation of vulnerable varieties, the unregulated movement of wheat grain, changes in pathogen virulence, the emergence of fungicide resistance, and a probable host jump, may result in severe crop losses and spread the disease to other major wheat-producing areas. As noted by Duveiller et al. [27], the potential for wheat blast to spread in Latin America remains high in susceptible regions of Mexico, Ecuador, and the Andean valleys (Figure 3). In addition to South America, the southeastern U.S. states, such as Louisiana, Mississippi, and Florida, are also at risk of wheat blast spread [28].

The first blast incidence outside South America and the most significant outbreak were documented in 2016 in Bangladesh, resulting from the intercontinental spread of the pathogen [4,29]. Although the initial outbreak of the disease was limited to only eight districts, it caused disastrous effects on local wheat production. Farmers were forced to burn their crops in the field, which had a detrimental impact on wheat production in the subsequent years. The total wheat-growing area impacted by blasts was 92,959 hectares in 2015–2016, which declined to only 47,278 hectares in 2016–2017 [32]. Likewise, during the 2017–2018 season, the total area of wheat cultivation in Bangladesh fell to 0.349 million hectares, representing just 79% of the preceding year’s figures and marking the lowest level in 30 years. Consequently, the areas under wheat cultivation in the blast-hit districts decreased by 52%. The disease continues to seriously threaten the potential for wheat cropping in Bangladesh, forcing farmers to switch to alternative cropping and reducing wheat yields by as much as 51% on average in affected fields [32]. Several studies employing prediction models have assessed the threat to wheat production in neighboring countries. These studies indicated that India, China, and Pakistan are areas of high risk for the further spread of wheat blast in the future [26,30]. The blast-sensitive regions encompass the densely populated and heavily farmed Indo-Gangetic Plain [33], which is the most important wheat–producing area in the region. It is anticipated that the blast-susceptible zones in this region will increase to 7 million ha, with an annual potential yield loss of 0.87–1.17 million tons [34]. Additionally, wheat cultivars grown in South Asia lack adequate blast resistance and face breeding challenges due to limited availability of resistance sources [26,31]. Collectively, these factors increase the wheat production system’s vulnerability to future outbreaks of wheat blast.

Wheat blast has also been found in Africa. The disease was first reported in a trial field in the Mpika district of northern Zambia, in Muchinga province in 2018 [35]. The occurrence and intensity of the disease were high due to weather conditions that supported the disease lifecycle and the reproduction of the pathogen. Similarly, in subsequent years, the disease was detected in the agricultural fields of the Mpika district, attributed to the hot, humid conditions [31]. The disease has since then been a significant menace to wheat cultivation in Zambia, particularly in rain-fed ecosystems. The broader importance of the disease in wheat production in Africa, however, remains unknown. A few experts believe that the disease could spread to other susceptible areas of Africa, such as Ethiopia, Kenya, and Congo, and cause potential crop losses [36]. Any potential spread of the disease to new areas would deal a significant blow to African wheat production, jeopardizing food security and the livelihoods of millions of people across the continent.

Given the belligerent particularities of the pathogen, wheat blast is a worrisome disease. One of the most significant concerns is its broad host range and its ability to evolve efficiently. MoT is able to infect a larger number of cultivated plants, such as barley, oat, rye, and forage grasses, compared with any of the twenty known pathotypes of *M. oryzae* [37]. MoT is a quick-acting fungal pathogen that can affect wheat ears, leading to significant yield loss within a few days [5]. The pathogen’s capacity to spread rapidly within and between countries via seeds, crop debris, and spores, and to persist within them over time, enhances the risk. Additionally, the Triticum pathotype exhibits greater genetic diversity than the other pathotypes [15]. These arrays of adaptations not only favor its transmission in agroecosystems but also hinder attempts to manage it [38]. Measures such as enforcing quarantine and biosafety protocols, as well as breeding more blast-resistant wheat varieties, can be primary tasks in preventing the growing threat of this disease [39].

**Table 1:** First report of the emergence of wheat blast across different wheat-growing countries and regions.

Country	Region	Year of Emergence	Severity of the Wheat Blast Outbreaks	Current Status of the Disease	References
Brazil	Paraná, Sao Paulo, Mato Grosso do Sul, Rio Grande do Sul, Minas Gerais, Goias, Brasília	1985–1993	Initial yield losses of 10–12%; in some areas, widespread outbreaks with yield losses up to 100% during epidemics	Widespread across all wheat-producing zones in Brazil, with recent epidemics in 2009 and 2012	[24,39]
Bolivia	Santa Cruz	1996	During the first epidemic in 1996, up to 100% yield loss in early-sown fields	Widespread across all wheat-producing zones in Bolivia, with a recent epidemic in 2014	[39,40]
Paraguay	Alto Parana, Itapua, Caaguazu, Caazapa, Canindeyu and Guaira	2002	Yield losses up to 80% in early-sown crops during the first epidemic	significant impact on major wheat production zones	[39,41]
Argentina	Chaco and Corrientes	2007	Limited impact initially	Presence in major wheat areas was noted, and concern grew after detection in the major wheat-producing province of Buenos Aires	[39,42]
USA	Kentucky	2011	Only a single spike was infected	Since then, the disease has not occurred.	[43]
Bangladesh	Kushtia, Meherpur, Chuadanga, Pabna, Jessore, Jhenaidah, Bhola, Barisal, Magura, Faridpur, and Rajshahi	2016–2017	First Asian outbreak affected ~15,000 ha with yield losses up to 51% in some districts	Spread to additional districts and is now present in all wheat-growing areas and affects wheat at various intensities—average yield loss of 15 to 24.5%	[4,5,29,44]
Zambia	Mpika district, Muchinga Province	2017–2018	Limited to the experimental field	Spread to the farmer’s field, but still with limited impact	[35]

## 4. Plant Immune System: An Overview

**Figure 4:**
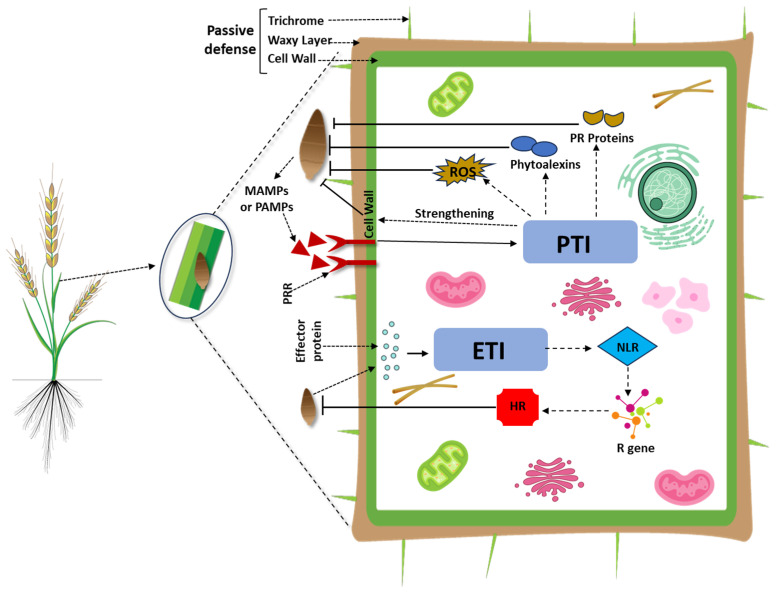
A schematic illustration of the plant immune response to wheat blast pathogen invasion. Plants utilize a multi-tiered immune system to defend against pathogenic microbes such as the wheat blast fungus. The first layer of defense comprises passive (pre-formed) structural barriers, including the plant cell wall, waxy cuticle, and trichomes, which physically obstruct pathogen entry. When these barriers are breached, the plant activates inducible (active) defense mechanisms. Recognition of PAMPs (or microbe-associated molecular patterns, MAMPs) by pattern recognition receptors (PRRs) initiates PAMP-Triggered Immunity (PTI). PTI responses include reinforcement of the cell wall, the generation of reactive oxygen species (ROS), the synthesis of phytoalexins, and the induction of pathogenesis-related (PR) proteins. However, pathogens can evade PTI by secreting effector proteins that suppress host immunity, leading to effector-triggered susceptibility (ETS). In response, plants deploy Effector-Triggered Immunity (ETI), mediated by intracellular nucleotide-binding leucine-rich repeat (NLR) proteins encoded by resistance (R) genes. These NLRs recognize pathogen effectors either directly or indirectly, triggering a more robust immune response, often characterized by a hypersensitive response (HR). This localized cell death helps contain the pathogen and limit its spread.

Plants defend themselves against attacks from a variety of organisms with complex defense mechanisms. There are two types of plant defense: pre-existing or passive defense and inducible or active defense, which confer immunity against pathogens. Structural defenses, such as plant cell walls, the waxy cuticle layer, and trichomes, which are always present on plants, hinder the entry and establishment of the pathogen, thus directly acting as a passive barrier [45]. The pre-existing defense systems adequately defend plants against the vast majority of the invading microbes. However, a few microorganisms acquire the ability to overcome passive or constitutive barriers in their host and become pathogenic [46]. To combat such pathogens, plants depend on more vigorous inducible defense systems. These systems are highly meticulous and are triggered only when plant cells detect a pathogen attack [13]. The successful detection of microbial elicitors, which are evolutionarily conserved molecular patterns referred to as MAMPs or PAMPs, occurs through Pattern Recognition Receptors (PRRs) based at the plant plasma membrane (Figure 4). This recognition activates downstream signaling pathways, initiating the plant’s first line of defense known as PTI [47]. The majority of plant PRRs are proteins, categorized as receptor-like kinases (RLKs), which are essential for activating plant immunity [48]. Interestingly, Damage-Associated Molecular Patterns (DAMPs), which include endogenous components of plant cells that are fragmented or damaged and released from dying cells, as well as ATP and High Mobility Group Box proteins, can initiate PTI or augment defense responses [49].

A number of defense responses, such as cell wall reinforcement, oxidative burst, phytoalexin production, and PR proteins induction, can result from recognition of MAMPs or PAMPs by PRRs. However, pathogen avirulence (AVR) genes frequently encode effector proteins that are secreted into plant cells and subvert PTI signaling, inducing effector-triggered susceptibility (ETS) [50]. In response to this ongoing evolutionary arms race, plants have evolved methods to counteract ETS. One such strategy is the incorporation of *R* genes that recognize specific effector proteins secreted by pathogens, activating a secondary defense mechanism known as ETI [13]. Extensive research has shown that *R* genes typically encode intracellular proteins containing nucleotide-binding leucine-rich repeats (NLRs) [51]. It is assumed that effective ETI occurs through the direct interaction (binding) of NLRs (receptors) with effectors produced by pathogens (ligands). This recognition induces a robust level of resistance, such as the hypersensitive response (HR) [47]. Moreover, a secondary interaction involving R-proteins and plant proteins is affected by effector proteins and can also lead to resistance. This assumption has been referred to as the “guard hypothesis” [13].

Although triggered by distinct PAMPs, PTI and ETI activate many of the same signaling cascades, such as the oxidative burst and the accumulation of PR proteins, which differ only in duration and intensity [47]. While HR has been regarded as a defining characteristic of ETI, it has also been shown that HR can be elicited by PAMP-triggered PTI [52,53]. Therefore, ETI has been considered a stronger form of PTI [54,55]. Moreover, both types of immune responses alter ion flux, redox status, and activate mitogen-activated protein kinase (MAPK) pathways, ultimately inducing gene expression for plant defense [47]. Increasing evidence also suggests that PTI- and ETI-mediated resistance is implicated not only in resistance specific to certain cultivars but also in resistance in non-hosts [56,57]. Therefore, PTI and ETI hold promise for crop improvement. However, the role and nature of their interaction regarding wheat blast remain unknown.

## 5. Roles of *Rmg* Genes in Wheat Blast Resistance

Deploying wheat blast resistance is the most justifiable and inexpensive method to control the disease. However, the number and sources of wheat blast-resistant genes are not only limited but have also been mostly investigated at the seedling stage. Again, the majority of these genes confer race-specific resistance, and their expression depends on the plant development stage, genetic background, and environmental conditions [12]. To effectively resist wheat ear blast, the resistant genes must be consistently expressed during the heading stage and remain effective even under elevated temperatures. These requirements make their deployment in wheat cultivar improvement programs challenging. So far, a total of 10 single major *Rmg* genes (Resistance to *M. grisea*) conferring resistance to MoT have been identified (Table 2). These genes include *RmgTd*(t), *Rmg1*(*Rwt4*), *Rmg2*, *Rmg3*, *Rmg4*, *Rmg5*, *Rmg6*(*Rwt3*), *Rmg7*, *Rmg8*, and *RmgGR119*. The majority of these genes render resistance to *Triticum* strains of *M. oryzae* (MoT strains/isolates). The gene *RmgTd*(t) was discovered in the tetraploid (*T. dicoccoides*) wheat variety “KU109” (Tat4), which demonstrated moderate resistance to a MoT isolate [58]. The gene is considered to be a hidden or concealed resistance gene, as it confers resistance by triggering an HR reaction in the mesophyll cells. Two additional genes, *Rmg2* and *Rmg3*, were mapped on chromosomes 7A and 6B, respectively, in the cultivar Thatcher of common hexaploid wheat (*Triticum aestivum*) against two MoT strains of *M. oryzae* [59]. These temperature-sensitive genes primarily confer resistance in seedlings, but are ineffective at elevated temperatures (around 25 °C) during the heading phase [60].

The dominant single gene, *Rmg7*, was identified in a tetraploid wheat variety, St24 (*T. dicoccum*, KU120), against the MoT isolate Br48 [60]. Two additional wheat cultivars, St17 (*T. dicoccum*, KU112) and St25 (*T. dicoccum*, KU122), were also found to possess this gene. Molecular cloning revealed that *Rmg7* is a variant of the *Pm4* gene positioned on chromosome 2AL, which confers resistance to wheat powdery mildew [69]. The gene is effective at both the seedling and heading stages [60]. Despite the efficacy of this gene being maintained at temperatures between 21 and 24 °C, it loses its resistance as the temperature increases above 26 °C [65]. Moreover, *Rmg7* has been overcome by existing MoT isolates [12].

Two other significant resistant genes, *Rmg8* and *RmgGR119*, were spotted in common wheat and Albanian Wheat Accession GR119, respectively [64,66]. *Rmg8* and *RmgGR119* are well known for their ability to maintain defense against the wheat ear infection by MoT and offer resistance at high temperatures [70]. Moreover, combining *Rmg8* with *RmgGR119* confers effective resistance to MoT isolates from Brazil and Bangladesh during the heading phase in laboratory settings [65,66,71]. This suggests that the two genes have significant potential for use in breeding programs to develop blast-resistant wheat cultivars. However, *Rmg8* and *RmgGR119* still need to be evaluated against the existing MoT isolates in practical field studies.

A number of genes regulating resistance to non-*Triticum* isolates of *M. oryzae* have been reported. For example, the two genes, *Rwt3* (syn. *Rmg6*) and *Rwt4* (syn. *Rmg1*), which provide resistance to *Avena* and/or *Lolium* isolates of *M. oryzae*, respectively, have been characterized in the common wheat cultivar Norin 4 [61,63]. Both genes reside in close proximity on chromosome 1D of common wheat [63], which indicates that these genes might have been co-introduced from *Aegiolops tauschii* into common wheat [72]. *Rwt3* is the NLR, encoding a nucleotide-binding leucine-rich repeat immune receptor, and *Rwt4* acts as a tandem kinase. These two genes, along with their avirulence (*Avr*) genes, are considered host-specificity barriers that restrict the pathogen from exploiting certain genotypes [73]. *Rwt3* is effective against the *Avena* and *Lolium* pathotypes because the *Avr* gene *PWT3* is present in both pathotypes, whereas *Rwt4* plays a significant role against the *Avena* pathotype only, as *PWT4* is found in this pathotype [74]. It has been observed that transforming a *Triticum* isolate of *M. oryzae* with *PWT4* suppresses resistance mediated by *Rmg8* [74]. Thus, introducing *Rmg8* into lines carrying *Rwt4* could be a strategy for developing high resistance against blast. This implies the importance of considering both host *R* genes and pathogen *Avr* genes when designing durable blast management strategies in wheat.

## 6. Role of 2NS Translocation and QTL Mapping for Wheat Blast Resistance

Apart from *Rmg* genes, a translocation segment and several QTLs for resistance to wheat blast have been detected on various wheat chromosomes. The 2NS translocation segment, derived from wild wheat *Aegilops ventricosa* and introgressed into the short arm of wheat chromosome 2A (2AS/2NS), provides one of the most important sources of head blast resistance in naturally occurring epidemic conditions [28]. In many wheat lines, the 2NS translocation is the original and predominant source of blast resistance and is strongly associated with greater resistance to MoT isolates. Recent studies in the field have shown that wheat resistance to MoT is quantitative and that 2NS translocation can explain a significant portion of the resistance variability across diverse environments [75]. Similarly, He et al. [70] reported the predominant effect of 2NS translocation on field wheat blast resistance, identifying six other minor QTLs for blast resistance on chromosomes 1AS, 2BL, 3AL, 4BS, 4DL, and 7BS. Follow-up studies with two additional biparental populations have recently been conducted, identifying major effects of the 2NS translocation as well as minor QTLs [76]. A GWAS conducted in 184 South Asian wheat genetic lines discovered major and stable effects of 2NS translocation on wheat blast resistance in the field, combined with some MTAs on the chromosomes 1BS, 2AS, 6BS, and 7BL [76]. A subsequent GWAS study in 187 South Asian wheat lines identified 40 markers associated with wheat blast resistance [77]. A total of 33(82.5%) were found on the 2NS chromosome arm, with one located in each of seven different chromosomes (3B, 3D, 4A, 5A, 5D, 6A, and 6B). GWAS with 1106 lines from CIMMYT breeders’ nursery also noted a large effect of the 2NS translocation region on field blast resistance and mapped other MTAs on chromosomes 3BL, 4AL, and 7BL [78]. Remarkably, over 80% of recently developed CIMMYT and Kansas (USA) breeding lines were observed to contain the 2NS segment, which is linked to resistance to multiple diseases and a potential for high yield [78,79]. Further supporting its role, Wu et al. [80] identified 58 significant SNPs within the 28.9 Mb region of 2NS, explaining 9.4–28.5% of the phenotypic variation. A QTL was found on chromosome 2AS that explained as much as 84.0% of the phenotypic variation due to MoT infection, demonstrating the strength of the 2NS translocation in conferring blast resistance [81].

**Figure 5:**
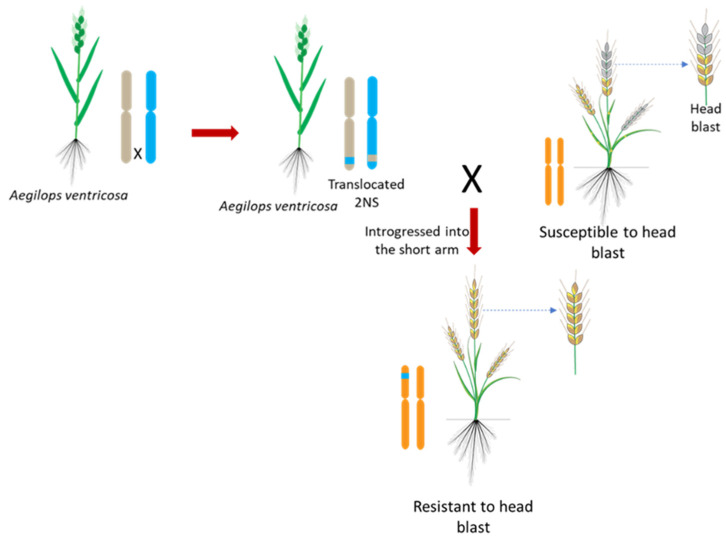
Molecular breeding strategy for developing wheat blast–resistant cultivars through introgression of the 2NS translocation segment from *Aegilops ventricosa*. The 2NS segment, incorporated into the short arm of wheat chromosome 2A (2AS/2NS), serves as the major and most reliable source of resistance to *Magnaporthe oryzae* pathotype *Triticum* (MoT) under field conditions. Repeated backcrossing combined with marker-assisted selection enables the efficient transfer and fixation of the 2NS segment into elite wheat backgrounds, thereby enhancing head blast resistance and ensuring the development of improved, high-yielding resistant cultivars.

On the contrary, a comprehensive study evaluating over 780 cultivated wheat and wild relative accessions in the field and greenhouse detected only 4 non-2NS spring wheat accessions from CIMMYT with demonstrable resistance to blast [82]. Nevertheless, the level of resistance observed was inadequate against some isolates, indicating the limited and unreliable nature of non-2NS resistance. This finding reinforces the status of the 2NS translocation as the most dominant and dependable major resistance locus. In contrast, loci located in other chromosomal regions have minimal phenotypic impact and are not consistently expressed [76]. Therefore, to enhance blast resistance against the contemporary MoT population, the 2NS chromosomal segment can be deployed in an elite wheat background through molecular breeding. This involves introgressing the 2NS chromosomal segment from *A. ventrocosa* into elite wheat backgrounds through repeated backcrossing coupled with MAS, which can accelerate the development of resistant cultivars against the current MoT (Figure 5).

Despite this, recent studies have reported some retrotransposon markers linked to significant and stable QTLs outside the 2AS/2NS translocation. For instance, the Brazilian cultivar BR 18-Terena has shown QTLs associated with blast resistance at the seedling stage on chromosomes 4A, 5A, and 2B, accounting for 17.8–19.6% of the phenotypic variance [83]. These findings suggest that although non-2NS loci generally contribute minor effects, they may still be valuable, especially when combined with other resistance mechanisms. Equally, while 2NS translocation has repeatedly demonstrated significant effects on wheat blast resistance (Table 2), overreliance on this single resistance source poses significant risks. The 2NS segment provides only partial resistance in adult plants, and field breakdown of 2NS-based resistance has been reported in South America [31].

## 7. Advances in Molecular Breeding for Wheat Blast Resistance

**Figure 6:**
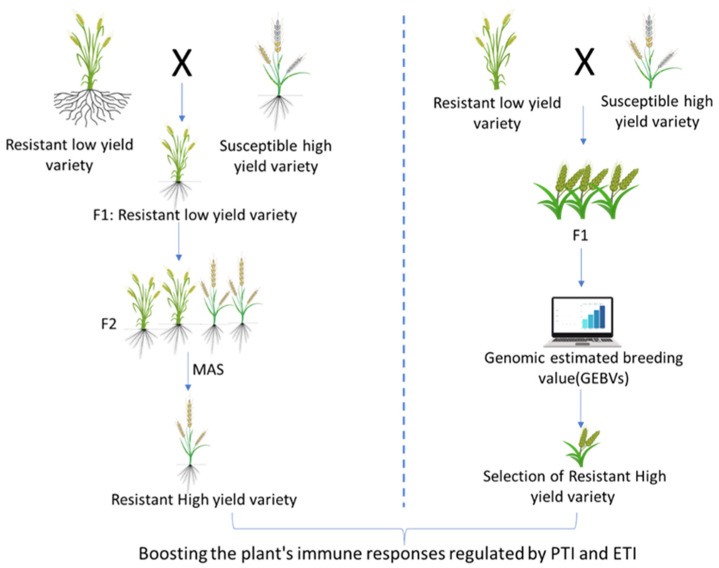
Comparative schematic representation of marker-assisted selection (MAS) and genomic selection (GS) in molecular breeding for wheat blast resistance. Both approaches accelerate the development of resistant cultivars by facilitating the precise incorporation of resistance genes and quantitative trait loci (QTLs). MAS targets specific markers linked to known resistance genes, whereas GS predicts genomic estimated breeding values using genome-wide marker data. Together, these tools enable rapid enhancement of wheat’s innate immune responses, integrating pattern-triggered immunity (PTI) and effector-triggered immunity (ETI) to confer durable blast resistance.

While conventional breeding faces several challenges in accelerating the development of blast-resistant wheat varieties, the application of molecular breeding tools and techniques can significantly improve its efficiency [31]. Marker-assisted selection (MAS) and genomic selection (GS), the two powerful tools in molecular breeding, can expedite the development of wheat varieties with long-lasting resistance by incorporating resistance genes and boosting immune responses regulated by PTI and ETI (Figure 6). The DNA-based, tightly linked molecular markers are crucial in introgressing blast-resistant genes into the candidate wheat cultivars. These markers enable breeders to select plants with desired traits by tracking their genetic makeup rather than relying solely on observable traits. MAS is particularly effective in reducing the breeding cycle and increasing the selection efficiency of desired genotypes (Figure 6). A wide variety of molecular markers are available for MAS, including single sequence repeats (SSR), Kompetitive Allele-Specific PCR (KASP), Diversity Arrays Technology sequencing (DArTseq), Single Nucleotide Polymorphisms (SNPs), and sequence-tagged sites (STS). These DNA markers have been effectively used in various QTL studies to map wheat blast resistance (Table 3). They are known for their high throughput, low cost, and accuracy [73].

Currently, SNPs are regarded as the most important markers for genetic mapping and Genome-Wide Association Studies (GWAS). SNP identification is typically performed through high-throughput sequencing. GWAS uses SNPs to identify genetic variants associated with diseases or resistance traits by comparing SNP frequencies in individuals with and without a specific condition. In wheat, several studies have identified SNP markers linked to 2NS translocation and various QTLs, enabling their use for MAS [70,85]. SNPs have also been used in several GWAS to identify the locations of genes in the wheat genome that contribute to blast resistance [76,78,84].

GS is particularly useful for predicting blast resistance using genome-wide markers rather than relying solely on phenotypic selection. This offers a tactical advantage over traditional phenotypic selection by enabling breeders to estimate genomic estimated breeding values (GEBVs) and select promising genotypes without extensive phenotyping [86]. This accelerates breeding cycles by enabling early selection of resistant lines, thereby reducing the time required to develop new cultivars (Figure 6). In GS, a “training population” comprising lines with known genotypes and phenotypes for the trait of interest is used to develop prediction models, which are then applied to genotyped-only individuals to estimate GEBVs. This strategy is particularly valuable in large breeding programs because it can accelerate selection for blast resistance in early generations that have undergone genotyping, despite the need to genotype many individuals.

Until now, GS has been proven successful at predicting quantitative resistance to wheat blast in large-scale screening [79,87,88], with advantages of increasing selection accuracy, reducing breeding cycles, and enhancing genetic gain [89,90]. For instance, the CIMMYT global wheat program screens 200–300 lines at the stage 3 yield trials for blast resistance, while approximately 9000 lines from the stage 1 yield trials are genotyped annually [75]. In this instance, employing the international nurseries as training populations, GS can estimate blast resistance in the early generation genotyped lines, resulting in considerable cost and resource savings. In this sense, GS may outperform MAS, as the same genotyping data can be exploited across multiple traits at early generations.

GS was evaluated for wheat blast phenotype at precision phenotyping platforms located in Quirusillas (Bolivia), Okinawa (Bolivia), and Jashore (Bangladesh) using three panels: (i) a 172-genotype diversity panel, (ii) 248 elite breeding lines, and (iii) 298 full-sib lines [91]. Two genomic prediction models, GBLUP and BayesB, were compared for accuracy against a fixed-effects model. The observed high prediction accuracies in the fixed-effects model can be attributed to markers tagging the 2NS translocation, which had a strong effect on blast across all panels. In areas where the 2NS translocation-dependent blast resistance is effective, a few markers tagging the translocation may adequately predict the blast resistance, making genome-wide markers unnecessary. Interestingly, MAS outperforms GS in this study, identifying the highest percentage (88.5%) of lines selected by phenotypic selection and eliminating the highest proportion (91.8%) of lines discarded by phenotypic selection.

While most studies demonstrate the effectiveness of MAS in selecting for 2NS translocation-mediated resistance, recent studies have identified and validated additional molecular markers suitable for MAS and variety screening (Table 4). Anh et al. [64] mapped the blast resistance gene *Rmg8* to the distal region of chromosome 2BL using F_3_ lines derived from S615 × Sch. The gene was flanked by the SSR markers Xwmc317 and Xbarc159, which can be used to track *Rmg8* in breeding populations. Similarly, *Rmg7* was localized to the distal region of chromosome 2AL in a cross between St24 and Tat14, with flanking SSR markers Xcfd50 and Xhbg327. Both sets of markers have been validated in segregating populations and can be applied for routine MAS to identify lines carrying these resistance alleles. Phuke et al. [85] conducted multi-environment genome-wide association studies (GWAS) across 350 Indian wheat genotypes in Bangladesh and Bolivia. While the 2NS translocation explained the largest proportion of phenotypic variation, several non-2NS SNPs were consistently associated with blast resistance. Notably, favorable alleles at 2B_180938790 (2BS), 5A_618682953 (5AL), and 7A_752501634 (7AL) were repeatedly detected across environments, and genotypes carrying all three showed a significantly lower blast index (<30%). These SNPs represent breeder-ready markers that can be applied for screening germplasm panels and pyramiding resistance alleles. He et al. [92] identified a major and stable QTL, Qwb.cim-7D, on chromosome 7DL, explaining up to 50.6% of the phenotypic variation in field trials across Bolivia and Bangladesh. The QTL was delimited to 619.90–625.61 Mb and validated with flanking KASP markers K3222157 and K1061589. These markers offer high-throughput, reliable selection tools for incorporating Qwb.cim-7D into elite breeding lines. Identification of validated SSR, SNP, and KASP markers linked to *Rmg7, Rmg8*, and novel QTLs provides valuable resources for MAS beyond the widely used 2NS segment. Their integration into breeding programs will facilitate the diversification of resistance sources, reduce vulnerability to 2NS breakdown, and accelerate the development of wheat cultivars with more durable blast resistance.

## 8. Genome Editing in Enhancing Wheat Blast Resistance

The hexaploid structure and the redundancy of gene functions in wheat make it quite laborious to employ genetic methods to select a specific phenotype and, in certain instances, unfeasible due to gene linkage or gene drag [93]. Genome-editing technologies have great potential to shorten this time and address linkage drag during the crop improvement process. Among the various platforms, Clustered regularly interspaced short palindromic repeat (CRISPR)-CRISPR-associated protein (CRISPR-Cas) is the most flexible, easy, and cost-effective strategy for specific genome modification [94,95]. It can alter DNA sequences specified by the engineered guide RNA without being limited to the three reading frames. This includes targeted mutagenesis methods such as gene knockouts, gene or allele replacements, and single-base substitutions. In this system, when double-strand DNA breaks (DSBs) occur due to CRISPR/Cas, repairs are carried out via the error-prone non-homologous end joining (NHEJ) pathway, the precise homology-directed repair (HDR) pathway, or a combination of both [96,97,98]. NHEJ is the primary repair mechanism for DSBs and typically results in random insertions and deletions (indels) at the site of chromosome reconnection [99]. As a result, most existing studies of plant genome editing rely on error-prone NHEJ-mediated spontaneous mutations and gene knockouts [100]. The presence of a DNA or RNA donor repair template (DRT) that contains homologous sequences adjacent to a DSB can induce HDR, leading to accurate gene replacement or insertion [101].

The wealth of genomic resources and molecular tools is now available, greatly enhancing crop improvement initiatives through CRISPR/Cas-mediated genome editing. Among these is the comprehensive wheat reference genome (IWGSC 2018), which serves as a foundational framework for understanding genetic variations. Furthermore, the newly established WheatGmap platform offers an impressive repository of over 3500 next-generation sequencing (NGS) datasets specifically for hexaploid wheat [102]. This extensive collection includes whole-genome sequences (WGS), whole-exome sequences (WES), and transcriptome deep-sequencing (RNA-seq) datasets. In addition to these resources, a high-resolution genomic variation map, created from the resequencing of 145 representative wheat cultivars from various historical periods [103], significantly enhances the identification of genes and the analysis of traits. These advancements can facilitate molecular breeders in choosing targets and assessing off-target effects in wheat genome editing.

Recently, numerous initiatives have been undertaken to use CRISPR/Cas to edit the wheat genome, aiming to improve agronomic traits such as grain yield and quality. Most of these genome-editing studies have utilized NHEJ to introduce loss-of-function mutations at specific gene loci in wheat plants [104]. For instance, *lipoxygenase* (*LOX*) is a crucial gene that plays multiple roles in wheat plants, including growth, development, and resistance to disease and wound stress [105]. However, knocking out *TaLOX2* using CRISPR-Cas has altered grain size and weight, and ultimately increased the storability of wheat grains [16]. The concurrent targeting of the three gibberellin-related *TaGASR7* genes, previously identified as regulators of grain size, resulted in a significant increase in the 1000-kernel weight [16]. The removal of the Phosphate 2 gene, *TaPHO2-A1*, has improved phosphorus uptake and increased grain yield in wheat when grown in low-phosphorus environments [106]. Knocking out the RING-type E3 ligase gene *TaGW2* has resulted in longer and wider wheat grains, ultimately leading to higher grain yield [107,108].

Some researchers have initiated efforts to improve the ability of wheat plants to withstand diseases using CRISPR/Cas9. Plants resistant to powdery mildew were developed in wheat through the editing of a *TaMLO* gene [109]. In a similar vein, CRISPR/Cas9 was used to eliminate an inhibitory regulator of the defense response to powdery mildew, *EDR1*, to obtain wheat plants with increased powdery mildew resistance [110]. A recent experiment using the CRISPR/Cas9 system to randomly delete a sequence that includes the start codon of a *TaHRC* gene in the wheat variety Bobwhite has led to resistance to Fusarium head blight, the most serious disease affecting quality and quantity [111]. These findings demonstrate the significant utility of CRISPR/Cas9 for enhancing disease resistance in wheat improvement programs.

**Figure 7:**
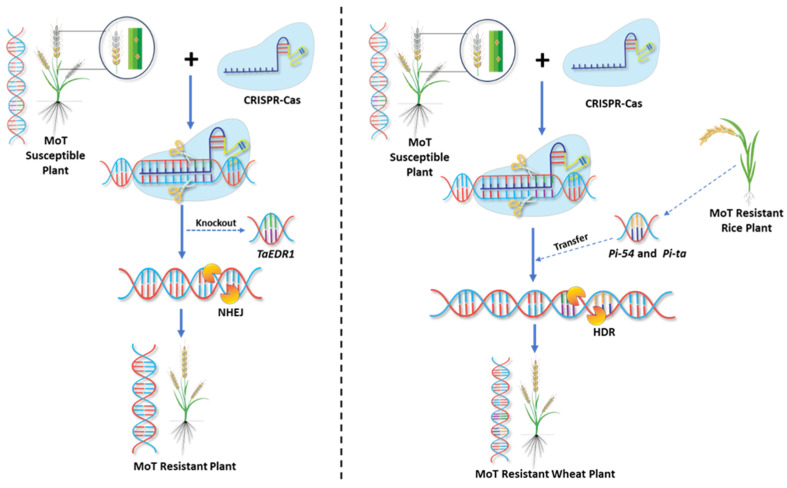
Schematic representation of CRISPR/Cas9-mediated gene editing approaches in wheat. (**left**) Gene knockout strategy: the Cas9 endonuclease, guided by a single guide RNA (sgRNA) targeting the gene of interest, introduces a double-strand break (DSB) in the DNA. Repair via the error-prone non-homologous end-joining (NHEJ) pathway generates insertions or deletions (indels) that disrupt gene (e.g., wheat susceptibility gene *TaEDR1*) function, producing knockout plants. (**right**) Gene knock-in strategy: Cas9/sgRNA-mediated DSBs are repaired via the homology-directed repair (HDR) pathway using a donor DNA template, enabling precise insertion or replacement of target sequences from another plant (e.g., rice blast resistant gene *Pi-54* and *Pi-ta* from a resistant rice genotype). The resulting plants exhibit the desired trait improvement or transgene integration.

CRISPR-based genome editing, using both gene knock-out and knock-in strategies, offers multiple approaches to improve wheat blast resistance (Figure 7). This includes both targeting susceptibility genes for disruption and the precise introduction of resistance alleles. The application of CRISPR/Cas9 can enable targeted edits in blast resistance (R) and susceptibility (S) genes, thereby enhancing the plant’s ability to recognize and combat MoT infections. Notably, targeted knockout of the wheat susceptibility gene *TaEDR1*, a negative regulator of immunity, has resulted in improved resistance to wheat blast without affecting yield [104]. Similarly, modifications in *ERF* transcription factors have strengthened wheat’s innate immune responses, demonstrating the potential of gene editing to enhance resistance [112]. Although specific S genes conferring susceptibility to MoT in wheat are still under investigation, analogous efforts in other pathosystems, for instance, the disruption of *TaHRC* to enhance resistance against Fusarium head blight, highlight the promise of targeting such genes in wheat blast [111]. Transcriptomic and comparative genomic analyses of wheat-MoT interactions are accelerating the identification of key targets for genome editing interventions [113]. CRISPR/Cas9 can also enable molecular stacking and functional enhancement of *R* genes. Transfer and optimization of blast-resistant genes from rice, such as *Pi-54* [114] and *Pi-ta* [115], by editing their wheat orthologs and synthetic gene design can confer broad-spectrum resistance to MoT. Engineering gene promoters to fine-tune gene expression for achieving optimal resistance against MoT through CRISPR/Cas9 also holds promise [93].

Apart from editing *R* and *S* genes, editing of regulatory genes involved in PTI and ETI also shows potential. Modifying key signaling components such as NPR1, WRKY transcription factors, or receptor-like kinases (RLKs) could enhance basal defense and amplify immune responses without significant trade-offs in yield or fitness. Such modification could trigger immune signaling cascades, providing durable resistance against MoT. In addition, multiplex CRISPR/Cas-based approaches are being explored to simultaneously target multiple genes involved in blast resistance, increasing the robustness of resistance mechanisms [116]. These highlight the potential of molecular breeding and genome editing in wheat blast resistance.

## 9. Development of Wheat Varieties for Blast Resistance

While achieving high levels of resistance remains a challenge, screening studies have identified a few moderately to highly resistant varieties (Table 5). In Brazil, several wheat cultivars exhibit varying degrees of resistance to blast disease. Notable resistant cultivars include BR 18-Terena, BR24, BRS 404, BRS201, BRS229, CD 113, MGS3 Brilhante, ORS 1401, ORS 1403, IPR 85, TBIO Sonic, TBIO Mestre, TBIO Sossego, and CD 116 [9,75,83,117,118]. Cultivars, such as Milan, Caninde 1“S”, and BR8, showed the highest levels of resistance to wheat blast fungus [119]. Recently, TBIO Triunfo, a wheat cultivar from Biotrigo Genética in Brazil, has been released and identified as moderately blast resistant [75].

In Bolivia, the wheat variety Milan was used in breeding initiatives to develop resistant strains, including Paragua CIAT, Sausal CIAT, and Milan3/Atila/Cimmyt3 [9,118]. These cultivars have shown a significant level of blast resistance. Additional research has identified several other cultivars in Bolivia, including Urubó, San Pablo, and AN-120, which have shown resistance to wheat blast in field conditions [120]. Similarly, in 2019, a new biofortified wheat variety, INIAF Okinawa, was released in Bolivia [121]. This variety possesses 2NS translocations, which are attributed to its blast resistance.

In Bangladesh, the Bangladesh Agricultural Research Institute (BARI), in collaboration with the International Maize and Wheat Improvement Center (CIMMYT), has developed and released a new zinc biofortified wheat variety named “BARI Gom 33” [122]. This is the first commercial wheat variety in Bangladesh to be resistant to wheat blast. The Bolivian variety INIAF Okinawa has been used to develop BARI Gom 33, which has demonstrated resistance to wheat blast in the laboratory and in field trials conducted in Jashore, Bangladesh, the hotspot of wheat blast. Furthermore, BARI Gom 33 has proven resistant in the United States Department of Agriculture (USDA) Agricultural Research Service (ARS) Laboratory in Maryland. The variety was also found to be moderately resistant to *Helminthosporium* leaf blight and leaf rust diseases and typically yields 5–8% more than existing wheat varieties in Bangladesh [122]. Because of these superior characteristics, the release of BARI Gom 33 for farmers marks a significant step toward resilient wheat cultivation in Bangladesh.

In India, several blast-resistant wheat varieties have been successfully developed and released, including MACS-6478, DBW-88, HD3249, DBW-252, and DBW-187 [121]. A significant number of these varieties incorporate the CIMMYT genotype Milan in their pedigree, where the 2NS translocation serves as the key resistance factor. Additionally, Japan has initiated breeding programs aimed at integrating genes for blast resistance into local premier varieties [66].

Given the scarcity of resistant sources and the limited genetic variability in wheat, mutation breeding has been explored to induce spontaneous genetic variation for the development of new blast-resistant varieties. In Bangladesh, some progress has been made in using gamma rays to induce mutations in wheat lines to obtain blast resistance [123]. In this study, BARI Gom-30 demonstrated superior blast resistance in the M2 generation. However, to release as a variety, the mutant lines must be progressed through several more generations and evaluated for their resistance against MoT. Another study titled “Disease Resistance in Rice and Wheat for Better Adaptation to Climate Change”, with the assistance of the FAO, is currently underway, involving researchers from 10 countries to identify MoT-resistant mutants [124]. Another study began investigating induced mutations in 2018, using the parental variety TBIO Toruk. After analyzing several thousand lines treated with two gamma-ray doses (250 and 300 Gy) and one chemical treatment (2% EMS), a few mutant lines were found to be resistant to MoT race 4-06 [75]. This suggests that in the face of limited availability of resistant resources, mutational breeding can make a significant contribution to the decisive efforts to develop blast-resistant cultivars.

**Table 5:** Commercial wheat varieties resistant to wheat blast across different wheat-growing areas.

Variety	Country	Resistance Level	Background	Reference
BARI Gom 33	Bangladesh	High	2NS	[122]
Borloug 100	Bangladesh, Bolivia, Nepal	High	2NS	[31]
BR 18-Terena	Brazil	High	Non 2NS	[83]
BR8	Brazil	High	2AS/2NS	[119]
BRS 229	Brazil	High	Non 2NS	[125]
Caninde 1“S”	Paraguay	High	2AS/2NS	[119]
Milan	South America	High	2AS/2NS	[119]
Paragua CIAT	Bolivia	High	-	[9]
Parapeti CIAT	Bolivia	High	-	[9]
BRS 120	Brazil	Moderate	2NS	[126]
BRS 220	Brazil	Moderate	2NS	[126]
BRS 49	Brazil	Moderate	2NS	[126]
Caninde 1	Paraguay	Moderate	2NS	[9]
CD 116	Brazil	Moderate	2NS	[126]
IAPAR 53	Brazil	Moderate	-	[126]
IPR 85	Brazil	Moderate	-	[9]
Itapua 75	Paraguay	Moderate	2NS	[127]
Motacu CIAT	Bolivia	Moderate	Non 2NS	[127]
Patuju CIAT	Bolivia	Moderate	Non 2NS	[127]
Sausal CIAT	Bolivia	Moderate	2AS/2NS	[127]

## 10. Potential Challenges and Opportunities

Despite significant progress in our comprehension of the genetic and molecular mechanisms underlying wheat blast resistance, developing blast-resistant cultivars faces significant challenges, including a lengthy time frame, limited resistant resources, and difficulties in selecting for quantitative resistance [23,128]. Additionally, screening many lines for blast resistance is complicated by the need for specific hotspot locations for phenotyping and limited evaluation capacity [5]. These challenges hinder the deployment of *R* genes that underpin ETI and the effective use of PTI to enhance resistance against the deadly wheat blast pathogen MoT. The fungus is rapidly evolving and highly aggressive, leading to a level of genetic variation that exceeds that of other pathotypes [31]. In particular, the widespread deployment of wheat *R* genes leads to the loss of function of the pathogen’s avirulent gene, resulting in genetic changes that allow the emergence of potentially new virulent MoT strains [129]. The new strains may increase infection in wheat plants and overcome single *R* gene-mediated host resistance within a few years of their deployment [39]. These phenomena limit the deployment of quantitative resistance mediated by a single R gene and necessitate the stacking or pyramiding of multiple genes to enhance durability [31]. Pyramiding *R* genes with components of PTI and ETI can reinforce plant defense and buffer against MoT variability.

There are other challenges, including difficulties in identifying broadly effective resistance genes. Modern wheat cultivars have a narrow genetic base resulting from a domestication bottleneck and highly selective breeding for key traits [130]. Moreover, the hexaploid wheat genome is large and complex [131]. This complexity poses significant challenges in the identification of robust and broadly effective *R* genes as well as in gene cloning, functional validation and precise introgression through conventional breeding or MAS.

Another challenge lies in the limited understanding of the component of PTI specific to wheat-MoT interactions. While PTI provides the first line of defense through PAMP, the wheat-specific PAMP receptors and their downstream signaling cascades have not been fully characterized [102]. This restricts their application in resistance breeding against MoT. On the other hand, ETI responses, often mediated by nucleotide-binding leucine-rich repeat (NLR) proteins, can lead to hypersensitive reactions and undesirable fitness costs [47]. Therefore, careful balance in their deployment is essential. Furthermore, understanding the interaction between PTI and ETI is crucial for developing strategies to build stronger, more durable resistance against a diverse pathogen population. However, the molecular mechanisms underlying crosstalk between the PTI and ETI pathways during MoT infection in wheat remain poorly understood [132]. This makes it difficult to harness these pathways synergistically for enhanced disease resistance against MoT.

Recent advances in integrated breeding approaches and biotechnological tools have created substantial opportunities to overcome these hurdles. The application of GWAS, high-throughput sequencing, and pan-genomics has accelerated the discovery of novel *R* genes and QTL associated with wheat blast resistance [70]. CRISPR/Cas genome editing technologies have emerged as powerful tools to edit susceptible genes, fine-tune the expression of resistant genes, and precisely stack multiple *R* genes without linkage drag [133]. Emerging concepts such as synthetic *R*-gene design, predictive modeling of pathogen effector evolution, and the application of machine learning in plant-pathogen interaction studies further enhance our ability to engineer durable resistance [134].

## 11. Conclusions and Future Perspectives

The escalating threat of wheat blast, now spreading beyond its initial epicenters in South America to regions such as Zambia and Bangladesh, underscores the urgent need for sustainable and durable resistance strategies in wheat. Host resistance remains the cornerstone of wheat blast management; however, currently available genetic resources beyond the 2NS translocation are limited. Although ten *R*-genes conferring blast resistance have been identified to date, their efficacy is often compromised by the emergence of new MoT strains, high-temperature sensitivity, and stage-specific expression.

Future progress on wheat blast resistance depends on broadening the genetic base and strengthening the multilayered immune system. Identifying and deploying novel *R* genes, particularly those with durability across diverse environments and developmental stages, will be crucial. Integrating broad-spectrum PTI with the rapid and robust *R* gene-mediated ETI offers a promising path toward lasting resistance. Harnessing allelic diversity from wild relatives can further enrich resistance reservoirs.

Advances in genomics and precision breeding tools are instrumental in transforming the landscape of resistance breeding. MAS, GS, GWAS, and CRISPR/Cas-mediated genome editing are revolutionizing resistance improvement by enabling the identification, stacking, and fine-tuning of defense genes. A synergistic approach, combining classical breeding, modern biotechnology, and deeper insights into host–pathogen interactions will be pivotal for developing wheat cultivars with durable, broad-spectrum, and climate-resilient blast resistance. In the face of rapidly evolving MoT populations and climate uncertainty, reinforcing plant defenses through integrated strategies represents the most promising frontier for sustainable wheat production.

## Figures and Tables

**Table 2:** Genes for resistance to wheat blast disease, their origin and efficacy against different pathogen isolates.

Gene	Wheat Species	Cultivars	Chromosome	Pathotype	Isolate	Efficacy	References
*RmgTd(t)*	*Triticum dicoccoides*	Ku109 (Tat4)	-	*Avena*, *Triticum*	A mutant progeny	Confer moderate resistance	[58]
*Rmg1(Rwt4)*	*T. aestivum*	Norin 4	1D	*Avena*	Isolate Br58	Confers resistance in seedlings and heads, but is temperature sensitive	[61]
*Rmg2*	*T. aestivum*	Thatcher	7A	*Triticum*	Isolate Br48	Confer resistance during the seedling stage; it is temperature sensitive	[59]
*Rmg3*	*T. aestivum*	Thatcher	6B	*Digitaria*	Isolate Br49	Provide high resistance even at elevated temperatures (26 °C)	[59]
*Rmg4*	*T. aestivum*	Norin 4	4A	*Digitaria*	Unkniwn isolate	Provide high resistance even at elevated temperatures (26 °C)	[62]
*Rmg5*	*T. aestivum*		6D	*Digitaria*	Unknown isolate	Confer resistance in seedlings and heads, but temperature-sensitive	[62]
*Rmg6(Rwt3)*	*T. aestivum*	Red Egyptian	1D	*Lolium*, *Eleusine*, *Avenae*	Ryegrass isolate TP2	Confer resistance at the heading stage, but ineffective at 26 °C	[63]
*Rmg7*	*T. dicoccum*	Norin 4	2A	*Triticum*	Br48	Confer resistance at the heading stage, but ineffective at 26 °C	[60]
*Rmg8*	*T. aestivum*	KU120 (St24), KU112 (ST17), KU122 (ST25)	2B	*Triticum*	Br49	Confer resistance at the heading stage and even at 26 °C	[64,65]
*RmgGR119*	Albanian Wheat	S-615	-	*Triticum*	Br50	Confer high resistance to all Triticum isolates tested	[66]
*Rwt1*, *Rwt2*, *Rwt5*	*T. aestivum* (implied)	Not specified	-	*Setaria*, *Oryzae*	-	Host-specificity barriers; recognize effectors (PWT3, PWT4)	[67,68]

**Table 3:** QTL studies in mapping for wheat blast resistance using various DNA markers.

QTL Number	DNA Markers ^a^	Mapping Population	Reference
QWbr.emt-2 ^a^	KASP and SSRs	Backcross population	[81]
QPag.emt-2 ^a^			
QWbr.emt-5B			
QWbr.emt-7B			
Loco 2AS	DArTSeq and STS	Backcross population	[76]
Loco 2DL			
Loco 7AL			
Loco 7DS			
Loco 2AS	SNP	Designed panel	[78]
Loco 3BL			
Loco 4AL			
Loco 7BL			
Loco 1AS	SNP	Designed panel	[70]
Loco 2BL			
Loco 3AL			
Loco 4BS			
Loco 4DL			
Loco 7BS			
Loco 2A	SNP	Designed panel	[84]
Loco 1BS	SNP and STS	Designed panel	[76]
Loco 2AS			
Loco 6BS			
Loco 7BL			
Loco 1A	SNP	Designed panel	[83]
Loco 2B			
Loco 4A			
Loco 5A			

^a^ SSR—single sequence repeat, KASP—Kompetitive Allele-Specific PCR, DArTseq—Diversity Arrays Technology sequencing, SNP—Single Nucleotide Polymorphism, STS—Sequence-tagged sites.

**Table 4:** Validated molecular markers linked to non-2NS wheat blast resistance genes and QTLs (excluding 2NS), recommended for marker-assisted selection (MAS).

Gene/QTL	Chromosome	Marker Type	Flanking/Associated Markers	Validation & Utility	Reference
*Rmg8*	2BL	SSR	Xwmc317–Xbarc159	Flanking SSRs identified via bulked segregant analysis; suitable for MAS in segregating and breeding populations	[64]
*Rmg7*	2AL	SSR	Xcfd50–Xhbg327	Flanking SSRs validated in segregating lines; suitable for tracking in variety screening	[64]
Non-2NS SNPs (GWAS)	2BS, 5AL, 7AL	SNP (DArTseq)	2B_180938790; 5A_618682953; 7A_752501634	Repeatedly detected across 12 multi-location trials; genotypes carrying all three alleles showed <30% wheat blast index	[85]
Qwb.cim-7D	7DL	KASP	K3222157–K1061589	Major QTL explaining up to 50.6% variation; KASP markers developed and validated for high-throughput MAS	[92]

## Data Availability

No new data were created or analyzed in this study. Data sharing is not applicable.
